# Activated STAT3 signaling pathway by ligature-induced periodontitis could contribute to neuroinflammation and cognitive impairment in rats

**DOI:** 10.1186/s12974-021-02071-9

**Published:** 2021-03-23

**Authors:** Yi Hu, Xu Zhang, Jing Zhang, Xinyi Xia, Huxiao Li, Che Qiu, Yue Liao, Huiwen Chen, Zhiyan He, Zhongchen Song, Wei Zhou

**Affiliations:** 1 Department of Periodontology, Shanghai Ninth People’s Hospital, College of Stomatology, Shanghai Jiao Tong University School of Medicine, National Clinical Research Center for Oral Diseases; Shanghai Key Laboratory of Stomatology & Shanghai Research Institute of Stomatology, 639 Zhizaoju Road, Shanghai, 200011 China; 2 Laboratory of Oral Microbiota and Systemic Diseases, Shanghai Ninth People’s Hospital, College of Stomatology, Shanghai Jiao Tong University School of Medicine, National Clinical Research Center for Oral Diseases, Shanghai Key Laboratory of Stomatology & Shanghai Research Institute of Stomatology, 115 Jinzun Road, Shanghai, 200125 China

**Keywords:** Ligature-induced periodontitis, Cognitive impairment, Neuroinflammation, STAT3 signaling pathway, APP processing

## Abstract

**Background:**

Increasing evidence suggests a causal link between periodontitis and cognitive disorders. Systemic inflammation initiated by periodontitis may mediate the development of cognitive impairment. Our study aims to investigate the effect of ligature-induced periodontitis on cognitive function and the role of signal transducers and activators of transcription 3 (STAT3) in this process.

**Materials and methods:**

Ligature-induced periodontitis was established, and the rats were treated intraperitoneally with/without the pSTAT3 inhibitor cryptotanshinone (CTS). Alveolar bone resorption and periodontal inflammation were detected by micro-computed tomography analysis and histopathological evaluation. Locomotor activity and cognitive function were evaluated by the open field test and the Morris water maze test, respectively. The activation of microglia and astrocytes in the hippocampus and cortex was assessed by immunohistochemistry (IHC). The expression of interleukins (IL-1β, IL-6, IL-8, IL-21) in both the periphery and cortex was evaluated by RT-PCR and ELISA. The expression of TLR/NF-κB and ROS cascades was evaluated by RT-PCR. The expression of pSTAT3 and the activation of the STAT3 signaling pathway (JAK2, STAT3, and pSTAT3) in the periodontal tissue and cortex were assessed by IHC and Western blot. The expression of amyloid precursor protein (APP) and its key secretases was evaluated by RT-PCR. The level of amyloid β-protein (Aβ) and the ratio of Aβ1-40/1-42 were measured via ELISA in the plasma and cortex while IHC was used to detect the level of Aβ1-42 in the brain.

**Results:**

In periodontal ligature rats, significant alveolar bone resorption and local inflammatory cell infiltration were present. Apparent increases in inflammatory cytokines (IL-1β, IL-6, IL-8, and IL-21) were detected in peripherial blood and brain. Additionally, spatial learning and memory ability was impaired, while locomotor activity was not affected. Activated microglia and astrocytes were found in the cortex and hippocampus, presenting as enlarged cell bodies and irregular protrusions. Levels of TLR/NF-kB, PPAR and ROS were altered. The STAT3 signaling pathway was activated in both the periodontal tissue and cortex, and the processing of APP by β- and γ-secretases was promoted. The changes mentioned above could be relieved by the pSTAT3 inhibitor CTS.

**Conclusions:**

Ligature-induced periodontitis in rats resulted in systemic inflammation and further abnormal APP processing, leading to cognitive impairments. In this progress, the activation of the STAT3 signaling pathway may play an important role by increasing inflammatory load and promoting neuroinflammation.

**Supplementary Information:**

The online version contains supplementary material available at 10.1186/s12974-021-02071-9.

## Introduction

Periodontitis is a chronic inflammatory disease that caused by plaque biofilm and damages the supporting tissues around the teeth [1]. As a product of interactions among bacterial pathogens, including their toxic factors, and host inflammatory responses, periodontitis has a multifactorial etiology with a marked inflammatory profile [2]. Despite leading to a poor oral health, there is increasing evidence that periodontal disease could be an important risk factor for systemic diseases through increasing the levels of systemic cytokines [3, 4].

Periodontitis shares epidemiological associations and underlying mechanisms with systemic chronic inflammatory disease [5]. Furthermore, common inflammatory cytokines have been shown to be increased in patients with chronic periodontitis and are capable of initiating and maintaining mechanisms associated with the development of chronic systemic diseases such as cognitive disorders [6].

Currently, investigations on periodontitis and cognitive disorders are still at the stage of epidemiological and clinical case-control studies; however, the underlying pathology has not been clearly clarified [7, 8]. Previous studies mostly used models established by a specific pathogen or its virulence factors, such as *Porphyromonas gingivali*s (*P. gingivalis*) [9, 10]. In our previous studies, we also demonstrated that the intraperitoneal injection as well as the topical application of *P. gingivalis*-LPS can cause cognitive impairment associated with brain inflammation via the NF-κB/TLR4 signaling pathway [11, 12]. A limitation of existing reports is the use of similar periodontitis models established by specific periodontal pathogen or its virulence factor application. It is necessary to develop other models, such as ligature-induced models, which is more similar to clinical periodontitis and may be more capable of elucidating the possible pathology [13]. The effect of ligature-induced periodontitis on cognitive function has not yet been investigated.

In the production of inflammatory cytokines and signaling molecules, signal transducer and activator of transcription (STAT) are one of the major regulatory elements [14]. Among them, STAT3 is critical for regulating the expression of cytokines, chemokines, and other mediators that can induce and maintain an inflammatory environment [15]. STAT3 signaling is also a major intrinsic pathway for inflammation for its ability to induce the expression of a large array of inflammatory mediators and its role as a core transcription factor in diverse immune responses [16]. STAT3 is a critical pathway for regulating the disorders encountered by the immune and inflammatory system [16]. As cognitive disorders are associated with neuroinflammation and STAT3 plays an important role in the course of inflammation, we hypothesized that STAT3 and its downstream pathway may play an active role in the association between cognitive impairment and ligature-induced periodontitis.

In the present study, periodontitis in SD rats was established by placing ligatures at the maxillary first molars and inflammatory load as well as cognitive function was evaluated. Periodontal inflammation and bone resorption similar to periodontitis were indicated by micro-CT and HE staining. The effects of ligature-induced periodontitis on cognitive function were evaluated by the MWM test. The relative expression of inflammatory factors was detected by RT-PCR and ELISA in both peripherial blood and brain. The activation of microglia and astrocytes in the brain was observed by IHC analysis. The levels of markers in TLR/NF-κB and ROS cascades were assessed by RT-PCR. To further explore the underlying mechanism, the activation of STAT3 and the processing of APP were assessed by RT-PCR, ELISA, WB, and immunohistochemistry (IHC).

## Materials and methods

### Animals

All experimental protocols were approved by the ethical committee of the Animal Care and Experimental Committee of Shanghai Jiao Tong University School of Medicine and were performed according to the guidelines from the EU Directive 2010/63/EU. Efforts were made to minimize suffering due to surgery and to reduce the overall number of animals we used.

**Fig. 1:**
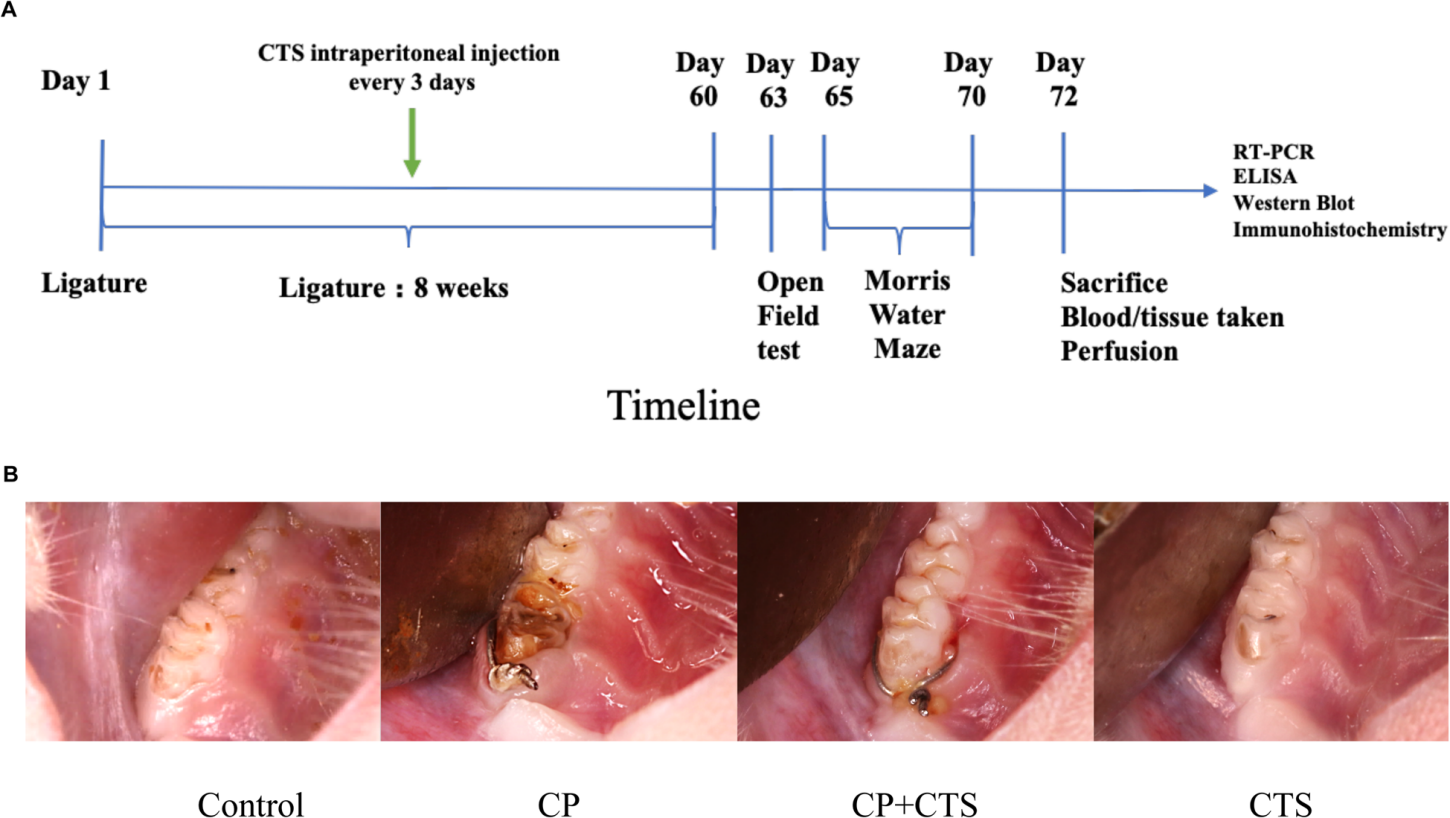
Timeline and intraoral photographs of periodontal ligature. **a** Timeline of the experiment. Periodontal ligature was placed for 8 weeks in the CP group and CP + CTS group, during which the CP + CTS group accepted CTS intraperitoneal injection every 3 days. After the last treatment, cognitive function was assessed by the OFT and MWM test. The underlying mechanism was further detected by RT-PCR, ELISA, WB, and IHC. **b** Representative intraoral photographs of 4 groups. Note the increased soft tissue edema around the ligature-treated molars compared to natural molar, and the edema was relived in the CP + CTS group

Ten-week-old male SD rats of specific pathogen-free (SPF) grade were provided by the Shanghai SIPPR-BK Laboratory Animal Co., Ltd. A standard housing of temperature (18–22 °C) and humidity (55–65%) with a 12-h light/dark cycle and free access to food and water was maintained during the experimental period. Rats were randomly allocated and 2 rats were housed per cage. The rats were given a minimum of 1 week to adapt to the new environment before the experiment. Thirty-two rats were divided into four groups: control, CP, CP + CTS, and CTS group. After anesthesia (intraperitoneal injection, 0.8 ml 5% chloral hydrate per 100 g weight), we opened the mouth of the rat using rubber bands. Then, 0.25-mm stainless steel wire ligatures were tied around the cervical subgingival region of the maxillary first molars to induce experimental periodontitis in the CP group and CP + CTS group. CTS was administrated by intraperitoneal injection (5 mg/kg) every 3 days. CTS possesses anti-inflammatory property, and the amount of CTS was calculated based on an earlier study [17]. Three days after the last administration, behavioral tests were performed to evaluate the cognitive function of the rats. Timeline and intraoral photographs could be found in Fig. 1a, b.

The 0.25-mm ligature wire was provided by the Department of Orthodontics, Shanghai Ninth People’s Hospital. CTS was purchased from Selleck (Shanghai, China) and dissolved basically according to Ryu et al. [18] as well as the instructions from the manufacturer with a few modifications (5% DMSO, 30%PEG300, and 5%Tween80 in volume fraction). The control group was given an equivalent volume of vehicle (5% DMSO, 30%PEG300, and 5%Tween80 in volume fraction), and no side effect was found.

### Measurement of alveolar bone resorption by micro-computed tomography (micro-CT)

The maxillae of rats were obtained to detect bone parameters by micro-CT. Fixed in 4% paraformaldehyde, the bone morphometry was assessed using Skyscan1172 (Bruker, Kontich, Belgium) with an accuracy of 18 μm. Parameters including bone volume percentage (bone volume/total volume, BV/TV), bone surface/volume ratio (BS/BV), and bone mineral density (BMD) were calculated.

### Assessment of periodontal inflammation responses by hematoxylin-eosin staining (HE staining)

For further histologic staining assay, the maxillae of rats were immersed with EDTA decalcifying solution (10% EDTA-2Na) at 4 °C for 4 weeks and the EDTA solution was changed every 3–5 days. After decalcification, hematoxylin-eosin (HE) staining assay was used to detect the inflammation response. Samples were then dehydrated through a serial alcohol gradient and embedded in paraffin wax blocks. Embed tissue in paraffin with the buccal side of the tooth facing towards the bottom of the micro mold, and the long axis of the teeth paralleling the short side of the cassette. Before immunostaining, 5-μm-thick tissue sections were dewaxed in xylene, rehydrated through decreasing concentrations of ethanol, and washed in PBS. Then, sections were stained with hematoxylin and eosin. After that, sections were dehydrated through increasing concentrations of ethanol and xylene.

### Isolation of peripheral blood mononuclear cells (PBMCs)

The peripheral blood from rats (5 ml per rat) was collected from the abdominal aorta in the presence of heparin as the anticoagulant. A 3 ml of the whole blood was diluted with sterile PBS of the same volume and gently resuspended. A 6 ml of the diluted whole blood fraction was overlaid onto a 3 ml of the Ficoll-Paque Plus (GE Healthcare Bio-Sciences Corp., Piscataway, NJ, USA) and then subjected to 800 g for 20 min at RT with the centrifuge brake “off.” Then, PBMC layers were washed twice with RPMI 1640 media by centrifugation at 1200 rpm for 5 min at 4 °C. After isolation, all samples were dissolved in Trizol reagent (Takara, Kusatsu, Shiga, Japan) for lysis of cells to extract RNA.

### RNA extraction and RT-PCR analysis

The RNA was extracted from PBMCs and homogenization of the cortex using Trizol reagent (Takara, Kusatsu, Shiga, Japan) and the Total RNA Kit (Omega Bio-Tek, Inc., Norcross, GA, USA), respectively. The extraction of RNA and the synthesis of cDNA were conducted according to Li et al. [19]. The purity and concentration of the samples were assessed by the Nanodrop Spectrophotometer (NanoDrop ND-1000; NanoDrop Technologies, Wilmington, DE, USA). The ratios of the absorbances at wavelengths of 260 and 280 nm were 1.8–2.0. A 20- to 30-mg tissue was used for RNA extraction, and a 1-μg RNA was used for RT-PCR. Subsequently, an RT-PCR assay was performed using SYBR Premix Ex Taq^TM^ (Takara, Kusatsu, Shiga, Japan) on a Roche LightCycler 480 Real-Time PCR Detection System (Roche, Basel, Switzerland) according to the manufacturer’s protocol. Data were then processed using the 2^-ΔΔCT^ method. All results were based on at least three independent tests, and the final results were expressed as normalized fold values relative to the control group. The sequences of genes including glyceraldehyde-3-phosphate dehydrogenase (GAPDH), interleukin-1β (IL-1β), IL-6, IL-8, IL-21, APP, amyloid precursor-like protein 1 (APLP1), APLP2, a disintegrin and metalloproteinase 10 (ADAM10), ADAM17, β-site APP cleaving enzyme 1 (BACE1), presenilin 1 (PS1), PS2, TLR2, TLR4, CD14, NF-κB, peroxisome proliferators-activated receptors (PPAR), superoxide dismutase (SOD), catalase (CAT), glutathione peroxidase 5 (GPx5), and their primer pairs were listed in Table 1.

**Table 1:** The sequences of genes and primer pairs

Target Gene	
GAPDH	Forward:5’-ACAGTCCATGCCATCACTGCC-3’
	Reverse:5’-GCCTGCTTCACCACCTTCTTG-3’
IL-1β	Forward:5’-AACCTGCTGGTGTGTGACGTTC-3’
	Reverse:5’-CAGCACGAGGCTTTTTTGTTGT-3’
IL-6	Forward:5’-GCCCTTCAGGAACAGCTATGA-3’
	Reverse:5’-TGTCAACAACATCAGTCCCAAGA-3’
IL-8	Forward:5’-CATTAATATTTAACGATGTGGATGCG-3’
	Reverse:5’-GCCTACCATCTTTAAACTGCACAAT-3’
IL-21	Forward:5’-GCTCCACAAGATGTAAAGGG-3’
	Reverse:5’-GTGCCTCTGTTTATTTCCTG-3’
APP	Forward:5’-AGAGGTCTACCCTGAACTGC-3’
	Reverse:5’-ATCGCTTACAAACTCACCAACT-3’
APLP1	Forward:5’-TCAGGTCTGCTGATCATGGGAGC-3’
	Reverse:5’-TGGGTGGGGAAGAGGACTTTATTG-3’
APLP2	Forward:5’-CAGAGCGACAGACCCTCATTC-3’
	Reverse:5’-TCTACTCGGGCCAAATGGGT-3’
ADAM10	Forward:5’-GCCTATGTCTTCACGGACCG-3’
	Reverse:5’-TGCCAGACCAAGAACACCATC-3’
ADAM17	Forward:5’-CAGGACGTAATTGAGCGGTTTT-3’
	Reverse:5’-ACGATGTTGTCTGCCAGAAACTT-3’
BACE1	Forward:5’-CGGGAGTGGTATTATGAAGTG-3’
	Reverse:5’-AGGATGGTGATGCGGAAG-3’
PS1	Forward:5’-GAGGAAGACGAAGAGCTGACAT-3’
	Reverse:5’-GAAGCTGACTGACTTGATGGTG-3’
PS2	Forward:5’-GAGCAGAGCCAAATCAAAGG-3’
	Reverse:5’-GGGAGAAAGAACAGCTCGTG-3’
TLR2	Forward:5’-GTACGCAGTGAGTGGTGCAAGT-3’
	Reverse:5’-GGCCGCGTCATTGTTCTC-3’
TLR4	Forward:5’-AGCCATTGCTGCCAACATCA-3’
	Reverse:5’-GCCAGAGCGGCTACTCAGAAAC-3’
CD14	Forward:5’-CTCAACCTAGAGCCGTTTCT-3’
	Reverse:5’-CAGGA TTGTCAGACAGGTCT-3’
NF-κB	Forward:5’-CAAGATCTGCCGAGTAAACC-3’
	Reverse:5’-TCGGAACACAATGGCCACTT-3’
PPAR	Forward:5’-AAGAACCTGAGGAAGCCA-3’
	Reverse:5’-AGCCACAAAAAGGGAAATG-3’
SOD	Forward:5’-CACTCTAAGAAACATGGCG-3’
	Reverse:5’-CTGAGAGTGAGATCACACG-3’
CAT	Forward:5’-ATGGCTTTTGACCCAAGCAA-3’
	Reverse:5’-CGGCCCTGGAGCATCTTGT-3’
GPx5	Forward:5’-CACCCCTCAGAGACTGTGGT-3’
	Reverse:5’-TTGACAGTGCTGACAGGAGC-3’

### Protein levels in the plasma and cortex measured by ELISA

Of the approximately 5 ml of the blood collected from the rats, 2 ml of the blood was collected in heparinized tubes for the measurement of plasma cytokines. After centrifuging (4 °C, 2500 rpm, 15 min), the plasma was immediately aliquoted into 1.5 ml cryogenic tubes and frozen at − 80 °C until use. For tissue, radioimmunoprecipitation assay (RIPA) lysis buffer (Beyotime, Beijing, China), 1% protease inhibitor cocktail (Sigma, St. Louis, MO, USA), and 1% PMSF (Beyotime, Beijing, China) were used to homogenize samples of the cerebral cortex. Protein qualification was performed by BCA Protein Assay Kit (Beyotime, Beijing, China). Levels of proteins were all measured by rat-specific ELISA kits. Equal amounts of protein were used in ELISA to measure levels of IL-1β (detection limits < 0.1 pg/ml; UBI, Sunnyvale, CA, USA), IL-6 (detection limits < 0.1 pg/ml; UBI, Sunnyvale, CA, USA), IL-8 (detection limits < 0.1 pg/ml; UBI, Sunnyvale, CA, USA), IL-21 (detection limits < 0.1 pg/ml; UBI, Sunnyvale, CA, USA), Aβ (detection limits < 1.0 ng/ml; UBI, Sunnyvale, CA, USA), Aβ1-40 (detection limits < 1.0 ng/ml; Enzyme-linked Biotechnology, Shanghai, China), and Aβ1-42 (detection limits < 0.1 ng/ml; Enzyme-linked Biotechnology, Shanghai, China) both in the plasma and cortex according to the manufacturer’s instructions.

### Open field test (OFT)

The open field in the present study consisted of a rectangular arena (530 mm × 478 mm), enclosed by a black wall, 590 mm in height (Mobile Datum, Shanghai, China). The test was initiated by gently placing a single rat in the middle of the arena, allowing the animal to move freely for 5 min while being recorded.

### Morris water maze (MWM) test

The MWM test was conducted in a round pool 160 cm in diameter and 55 cm in depth (Mobile Datum, Shanghai, China). The pool was filled with water made opaque with white non-toxic water-based tempura paint. The water temperature was controlled to remain with a range equivalent to that of room temperature (22 ± 1 °C). The pool was divided into 4 quadrants: northeast (NE), northwest (NW), southeast (SE), and southwest (SW) at equal distances on the rim. The platform was placed in the center of SW quadrant and submerged 2.5 cm beneath the water surface; it remained in the same position throughout the learning trials and was removed from the pool during the probe test. A video-tracking system (Shanghai Jiliang Software Technology Co., Ltd.) was used to monitor and record the swimming activity of the rats. The rats should have learned to use the visual tips around the pool to find the hidden platform within 90 s; otherwise, it would be gently guided to the platform and allowed to re-orient for an additional 10 s. Each rat was trained four times per day with 30 s of rest per training interval. To examine spatial reference memory, a probe test was carried out on the sixth day when the platform was removed from the pool and each rat was placed into the water at the two quadrants furthest from the platform used on days 1–5, being allowed to navigate freely for 60 s.

### Western blot (WB)

The samples of the periodontal tissue and cerebral cortex of rats in four groups were homogenized and lysed by RIPA containing 1% protease inhibitor cocktail and 1% PMSF (Beyotime, Shanghai, China). Equal amounts of protein were separated by SDS polyacrylamide gel electrophoresis and transferred onto PVDF membrane blocked with 5% skimmed milk as previously described [19]. A pre-stained protein marker (Thermo Fisher Scientific, MA, USA) was run in parallel to detect the molecular weight of proteins. GAPDH was used as a protein loading control according to its high and constant expression in most tissues and cell types, earning the gene and its protein housekeeping status. The proteins were probed with appropriate antibodies including anti-JAK2 (Rabbit mAb, 1:500, 3230 T; Cell Signaling Technology, USA), anti-STAT3 (Rabbit mAb, 1:1000, 4904 T; Cell Signaling Technology, USA), anti-phosphor STAT3-Tyr705 (Rabbit mAb, 1:200, 9145S; Cell Signaling Technology, USA), and anti-GAPDH (Rabbit mAb, 1:1000, AB-P-R001, Goodhere Biotechnology Co., Hangzhou, China). The data were quantified by ImageJ 1.51j8.

### Immunohistochemistry (IHC)

Rats were deeply anesthetized with 5% chloral hydrate and were perfused transcardially with 200 mL 0.9% saline (4 °C) before removal of the brain. One hemisphere was placed in 4% paraformaldehyde overnight at 4 °C, after which paraffin sections were prepared. This procedure was basically consistent with the previous study [11]. Briefly, brain sections were incubated with 3% H_2_O_2_ in methanol, blocked with 10% goat serum and incubated overnight at 4 °C with the following primary antibodies: ionized calcium-binding adaptor molecule 1 (Iba1) (Goat pAb, 1:400, ARG63338; Arigo Biolaboratories, Hsinchu City, Taiwan, China), GFAP (Rabbit pAb, 1:400, ab7260; Abcam), anti-beta Amyloid1-42 antibody (1:50, ab10148; Abcam), and anti-phosphor STAT3-Tyr705 (Rabbit mAb, 1:1000, 9145S; Cell Signaling Technology, USA). After being washed, the sections were incubated with biotinylated goat anti-rabbit or goat secondary antibody (1:200; Vector Laboratories, Burlingame, CA, USA). After being rinsed with PBS, streptavidin-labeled peroxidase was added and left for 30 min. This was followed by further rinsing, after which newly prepared 3,3′-diaminobenzidine (DAB) solution was added, and the mixture was left for the reaction to develop. The sections were dyed with hematoxylin and dipped in 1% hydrochloric acid in alcohol for differentiation. They were then washed in ammonia and stained blue, after which they were rinsed with water. Omission of the primary antibodies was used as negative controls (Supplementary Material 1).

Images were obtained with LAS V4.10 on a Leica camera (Leica DMi8). The shape analysis of microglia and astrocytes (including the endpoints and process length) were evaluated via ImageJ according to previous studies [20, 21]. The numbers of inflammatory cells and Aβ1-42 positive cells were determined by counting positive cells via ImageJ. At least three sections were analyzed per rat, and the average of the individual measurements was used to calculate group means.

### Statistical analysis

All data are presented as the mean ± standard error of the mean (SEM). *P* values were calculated with GraphPad Prism software. For the Morris water maze experiments, the escape latency during the spatial learning tests was determined by a two-way ANOVA. All of the other experiments were analyzed using one-way ANOVA. An analysis of variance was performed using Turkey’s post hoc multiple comparison test. A value of *p* < 0.05 was indicative of statistical significance.

## Results

### Assessment of alveolar bone resorption and periodontal inflammation

Maxillae were visually assessed for soft tissue differences between control and ligature groups. In the control group, the soft tissues showed the typical clinical presentation observed in healthy murine oral mucosa, which was served as a baseline (healthy tissue). However, in the CP group, the tissues appeared edematous (Fig. 1).

**Fig. 2:**
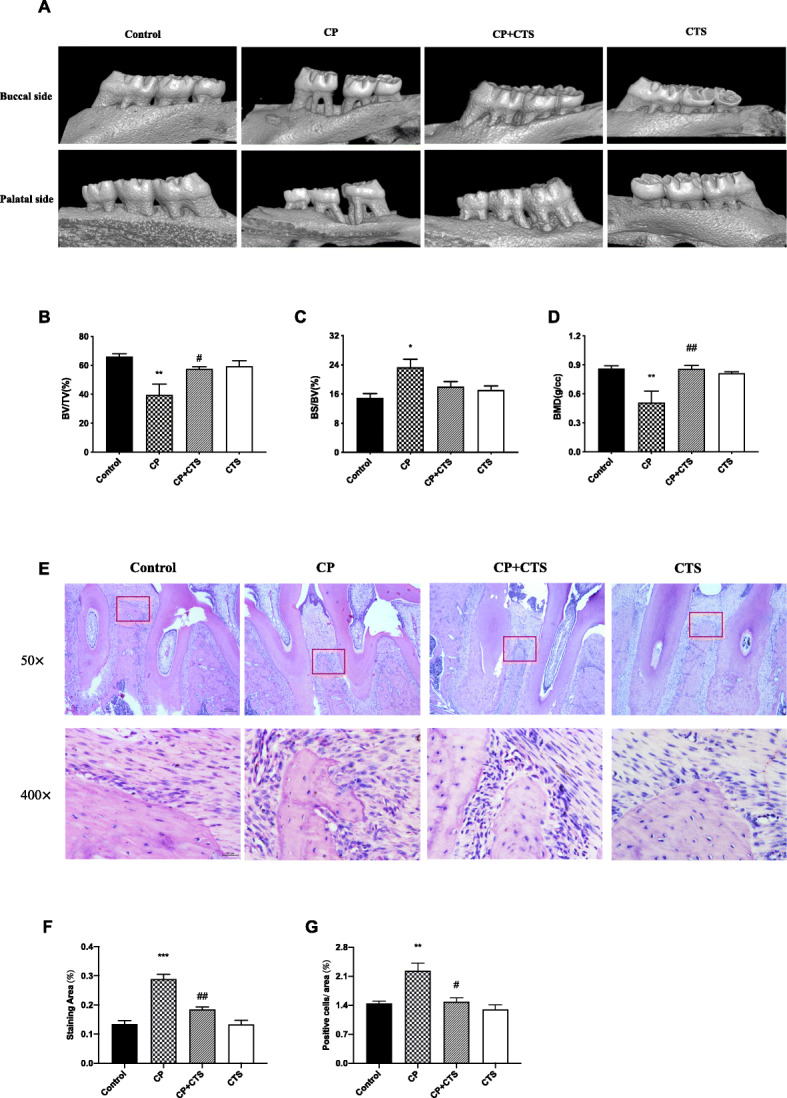
Effects of periodontal ligature on the periodontal tissue. **a** Images captured by micro-CT scanning of the molar region. Bone density analysis of the alveolar bone: **b** BV/TV (*n* = 4, *F* = 7.062, *p* = 0.0054), **c** BS/BV (*n* = 4, *F* = 5.488, *p* = 0.00131), and **d** BMD (*n* = 4, *F* = 7.323, *p* = 0.0048). After decalcification, hematoxylin-eosin (HE) staining assay was used to detect the inflammation response in maxillae of rats (**e**). Microscope images were at a magnification of ×100 and ×400 (bar = 200 μm and 50 μm, respectively). Quantified analysis conducted by ImageJ: **f** staining area (*n* = 3, *F* = 33.19, *p* < 0.0001) and **g** number of cells/area (*n* = 3, *F* = 11.72, *p* = 0.0027). (*n* = 3–4 per group, one-way ANOVA, **p < 0.01 and ***p < 0.001 compared to the control group; #*p* < 0.05 and ##*p* < 0.01 compared to the CP group)

As shown in Fig. 2a, posterior maxillary bone loss was observed in the CP group. BV/TV and BMD were decreased while BS/BV was increased in the CP group and was reversed by CTS (Fig. 2b–d). Further, HE staining suggested that periodontal ligature led to a decrease in alveolar bone height, irregular bone surface, and infiltration of inflammatory cells (Fig. 2e). Staining area and number of cells/area were evaluated by ImageJ (Fig. 2f, g). All of these changes, which were partly alleviated by CTS, suggested that periodontal inflammation and bone resorption similar to periodontitis could be induced by periodontal ligature.

### Effects of ligature-induced periodontitis on the level of inflammatory cytokines in the peripheral blood

**Fig. 3:**
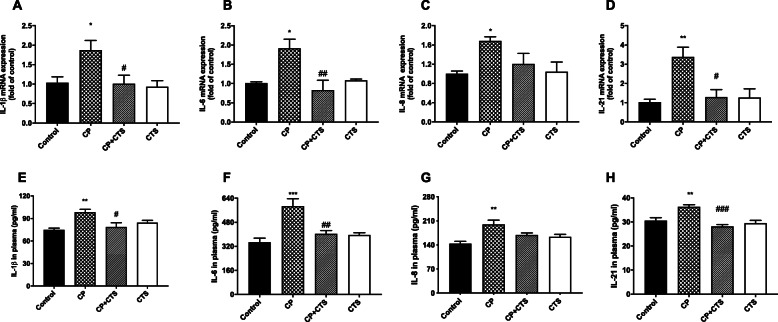
Effects of ligature-induced periodontitis on inflammatory cytokines in PBMCs. Periodontal ligature induced high expression of IL-1β, IL-8, IL-6, and IL-21 in PBMCs, while these changes were alleviated by CTS. RT-PCR was performed to detect the mRNA expression of cytokines in each group: **a** IL-1β (*n* = 4, *F* = 4.963, *p* = 0.0182), **b** IL-6 (*n* = 4, *F* = 7.600, *p* = 0.0041), **c** IL-8 (*n* = 4, *F* = 3.889, *p* = 0.0374), and **d** IL-21 (*n* = 4, *F* = 7.568, *p* = 0.0042). ELISA was performed to detect the protein expression of these cytokines in plasma in each group: **e** IL-1β (*n* = 5, *F* = 6.460, *p* = 0.0045), **f** IL-6 (*n* = 5, *F* = 11.09, *p* = 0.0003), **g** IL-8 (*n* = 5, *F* = 7.202, *p* = 0.0028), and **h** IL-21 (*n* = 5, *F* = 12.18, *p* = 0.0002). (*n* = 4–5 per group, one-way ANOVA, *p < 0.05, **p < 0.01, and ***p < 0.001 compared to the control group; #*p* < 0.05, ##*p* < 0.01, and ###*p* < 0.001 compared to the CP group)

RT-PCR assays were performed to evaluate the mRNA expression of inflammatory cytokines including IL-1β, IL-6, IL-8, and IL-21 in PBMCs (Fig. 3a–d). The protein levels of IL-1β, IL-6, IL-8, and IL-21 in the plasma were detected by ELISA (Fig. 3e–h). The CP group showed significant increases in the expression of the above cytokines on both mRNA and protein levels, and these increases were reversed by CTS. Upregulated expression of these inflammatory factors was induced by ligature-induced periodontitis and significantly prevented by CTS.

### Effects of ligature-induced periodontitis on locomotor activity and spatial learning and memory

**Fig. 4:**
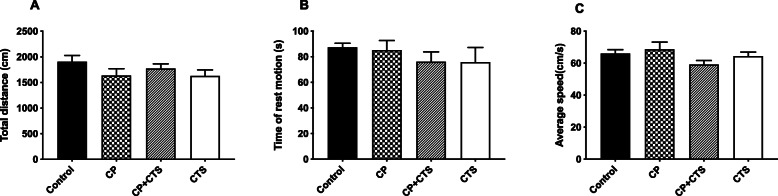
Effects of ligature-induced periodontitis on animal locomotor activity. The open field test (OFT) was used to evaluate the locomotor activity of rats after the final administration. **a** total distance (*F* = 1.327, *p* = 0.2937), **b** time of rest motion (*F* = 1.280, *p* = 0.3084), and **c** average speed (*F* = 1.728, *p* = 0.1934). Overall, no significant differences were observed between groups in OFT. (*n* = 6 per group, one-way ANOVA, *p* > 0.05)

The OFT was used to assess whether periodontal ligature or the administration of CTS could affect the spontaneous activity of rats. Behavior parameters, including the total distance covered (Fig. 4a), time of rest (Fig. 4b), and average speed (Fig. 4c), were not significantly different among the groups. Behavioral performance indicated that the locomotor activity of the rats was not affected by neither periodontal ligature nor CTS.

**Fig. 5:**
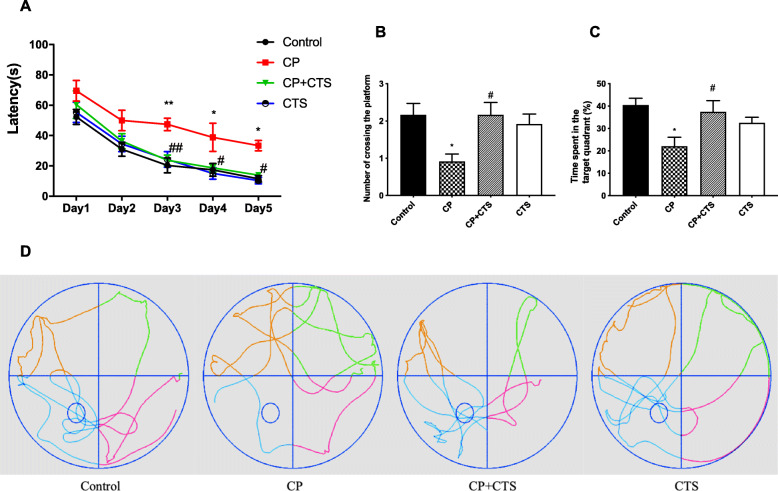
Effects of ligature-induced periodontitis on learning and memory. The Morris Water Maze test (MWM) was conducted to assess the learning and memory ability: **a** latency to find the platform during the acquisition phase of the MWM test (*n* = 6 per group, two-way ANOVA, *F* = 18.08, *p < 0.05, and **p < 0.01 compared to the control group, #*p* < 0.05, and ##*p* < 0.01 compared to the CP group), **b** number of platform crossings in the target quadrant (*F* = 4.435, *p* = 0.0152), **c** percentage of time spent in the target quadrant (*F* = 4.600, *p* = 0.0132), and **d** the typical trajectories. The platform is represented by an open circle. (*n* = 6 per group, one-way ANOVA, *p < 0.05 compared to the control group; #*p* < 0.05 compared to the CP group)

The MWM test was conducted to assess whether ligature-induced periodontitis could affect the learning and memory of rats. Over the 5-day training period, the latency of all groups decreased chronologically (Fig. 5a). The escape latency of the CP group was evidently longer than that of the control group after the 5-day training period. No significant differences were observed between the control group, the CP plus CTS group, and the CTS group. The platform was removed on the sixth day, and the following parameters were assessed in the probe test: the number of the platform crossed and the percentage of time spent in the target quadrant. Both of these parameters which were significantly reduced in the CP group (Fig. 5b, c). During the probe test, the rats in the control group learned to navigate directly to the quadrant that had contained the hidden platform. However, this behavior was strikingly compromised in the CP group (Fig. 5d). The abovementioned changes in the CP group could be reversed by CTS. The results of the behavioral tests revealed that ligature-induced periodontitis may be an important risk factor for learning and memory impairment.

### Effects of ligature-induced periodontitis on microglia and astrocytes

**Fig. 6:**
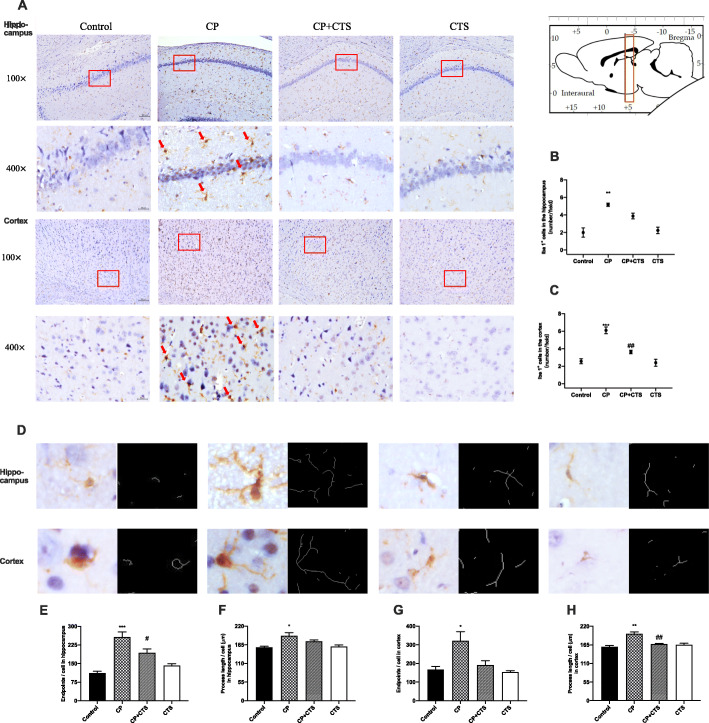
Effects of ligature-induced periodontitis on microglia in the hippocampus and cortex. Histopathological analysis of brain sections was performed using immunohistochemistry. The detected regions were illustrated in sagittal section of the brain. Activated microglia (with irregular protrusions) were attenuated by pre-treatment of CTS. Microglia were visualized with Iba1 (**a**) (red arrows). Quantification of Iba1 levels in the hippocampus and cortex were shown: **b** Iba1 positive cells in hippocampus (number/field) (*F* = 15.870, *p* = 0.0010), **c** Iba1-positive cells in the cortex (number/field) (*F* = 26.740, *p* = 0.0002). The example cells (**d**) and quantification of microglia were shown: **e** endpoints in the hippocampus (*F* = 21.080, *p* = 0.0004), **f** process lengths in the hippocampus (*F* = 7.370, *p* = 0.0109), **g** endpoints in the cortex (*F* = 7.008, *p* = 0.0125), **h** process lengths in the cortex (*F* = 15.020, *p* = 0.0012), (100× and 400×, bar = 200 μm and 50 μm, respectively). (*n* = 3 per group, one-way ANOVA, *p < 0.05, **p < 0.01, and ***p < 0.001 compared to the control group; #*p* < 0.05 and ##*p* < 0.01 compared to the CP group)

**Fig. 7:**
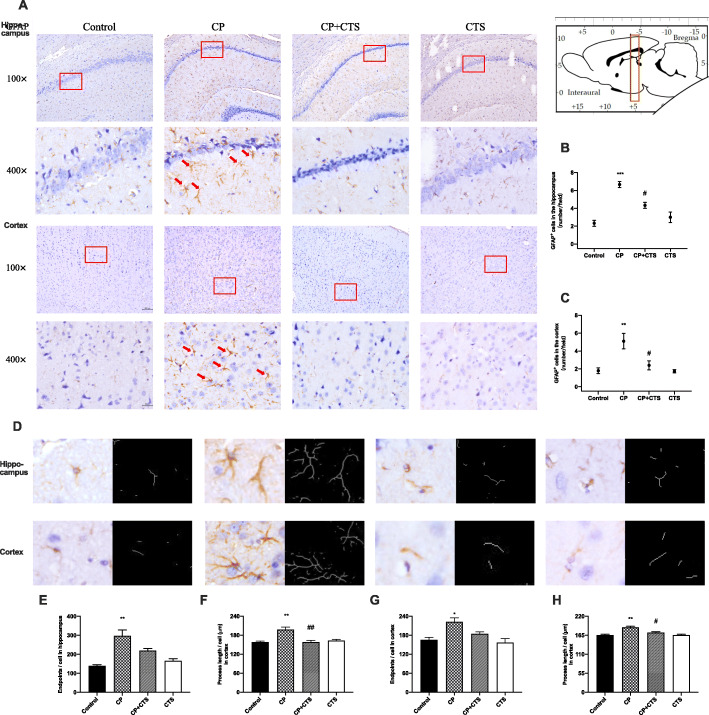
Effects of ligature-induced periodontitis on astrocytes in the hippocampus and cortex. Histopathological analysis of brain sections was performed using immunohistochemistry. The detected regions were illustrated in sagittal section of the brain. Activated astrocytes (with irregular protrusions) were attenuated by pre-treatment of CTS. Astrocytes were labeled by GFAP (**a**) (red arrows). Quantification of GFAP levels in the hippocampus and cortex were shown: **b** GFAP-positive cells in the hippocampus (number/field) (*F* = 21.940, *p* = 0.0003), **c** GFAP-positive cells in the cortex (number/field) (*F* = 9.016, *p* = 0.0060). The example cells (**d**) and quantification of microglia were shown: **e** endpoints in the hippocampus (*F* = 15.080, *p* = 0.0012), **f** process lengths in hippocampus (*F* = 13.760, *p* = 0.0016), **g** endpoints in the cortex (*F* = 7.640, *p* = 0.0098), **h** process lengths in the cortex (*F* = 16.080, *p* = 0.0009), (100× and 400×, bar = 200 μm and 50 μm, respectively). (*n* = 3 per group, one-way ANOVA, *p < 0.05 and **p < 0.01 compared to the control group; #*p* < 0.05 and ##*p* < 0.01 compared to the CP group)

As shown in Figs. 6 and 7, microglia (Fig. 6a–c) and astrocytes (Fig. 7a–c) were observed to be activated in the CP group. In both the hippocampus and cortex, activated microglia (labelled by Iba) with irregular protrusions and bushy, swollen cell bodies were found in the CP group (Fig. 6d–h). Relative increase in endpoints and irregular protrusions could be found in activated astrocytes, which were positively stained for GFAP (Fig. 7d–h). Changes mentioned above could be partly reversed by the administration of CTS. These findings indicated that ligature-induced periodontitis might play an important role in neuroinflammation.

### Effects of ligature-induced periodontitis on interleukins in the cortex

**Fig. 8:**
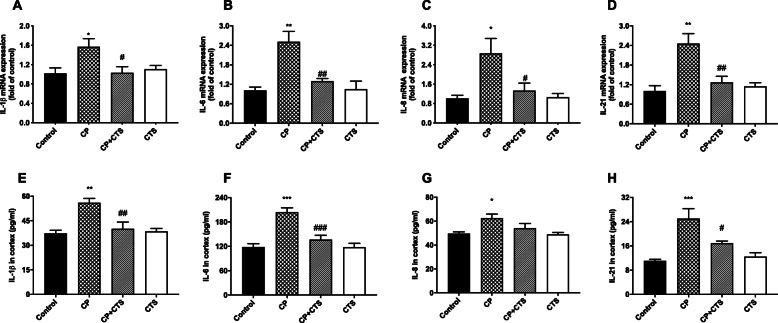
Effects of ligature-induced periodontitis on inflammatory cytokines in the cortex. RT-PCR and ELISA were performed to detect mRNA and protein levels of inflammatory cytokines (IL-1β, IL-6, IL-8, and IL-21) in the cortex. Periodontal ligature induced high expression of inflammatory factors on both mRNA and proteins in comparison to the control group, while these changes were prevented by CTS. The mRNA level of cytokines in the plasma in each group: **a** IL-1β (*n* = 4, *F* = 4.474, *p* = 0.0250), **b** IL-6 (*n* = 4, *F* = 11.3, *p* = 0.008), **c** IL-8 (*n* = 4, *F* = 5.979, *p* = 0.0098), and **d** IL-21 (*n* = 4, *F* = 10.83, *p* = 0.0010). The protein level of cytokines in cortex in each group: **e** IL-1β (*n* = 5, *F* = 9.945, *p* = 0.0006), **f** IL-6 (*n* = 5, *F* = 17.13, *p* < 0.0001), **g** IL-8 (*n* = 5, *F* = 4.744, *p* = 0.0149), **h** IL-21 (*n* = 5, *F* = 12.36, *p* = 0.0002). (*n* = 4–5 per group, one-way ANOVA, *p < 0.05, **p < 0.01, and ***p < 0.001 compared to the control group; #*p* < 0.05, ##*p* < 0.01, and ###*p* < 0.001 compared to the CP group)

RT-PCR assays were performed to determine the mRNA expression of IL-1β, IL-6, IL-8, and IL-21 in the cortex (Fig. 8a–d). In addition, ELISA was used to detect protein levels (Fig. 8e–h). Both the mRNA and protein expression of IL-1β, IL-6, IL-8, and IL-21 in the cortex were significantly higher in the CP group than in the control group. In comparison to the CP group, the CP plus CTS group showed a significant reduction. High gene and protein expression of these inflammatory factors in the cortex were stimulated by ligature-induced periodontitis and could be prevented by CTS.

### Effects of ligature-induced periodontitis on TLR/NF-κB, PPAR, and ROS

**Fig. 9:**
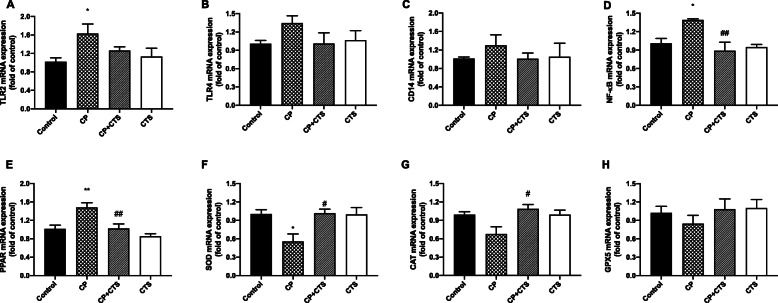
Effects of ligature-induced periodontitis on mRNA level of TLR/NF-κB and ROS cascades. mRNA levels of markers in TLR/NF-κB and ROS cascades were assessed by RT-PCR: **a** TLR2 (*n* = 6, *F* = 3.128, *p* = 0.0486), **b** TLR4 (*n* = 6, *F* = 1.423, *p* = 0.2656), **c** CD14 (*n* = 6, *F* = 0.4886, *p* = 0.6941), **d** NF-κB (*n* = 4, *F* = 7.134, *p* = 0.0052), **e** PPAR (*n* = 5, *F* = 9.699, *p* = 0.0007), **f** SOD (*n* = 6, *F* = 5.426, *p* = 0.0068), **g** CAT (*n* = 6, *F* = 4.547, *p* = 0.0138), and **h** GPx5 (*n* = 6, *F* = 0.6873, *p* = 0.5703). TLR2, NF-κB, and PPAR expression were significantly upregulated by periodontal ligature while SOD and CAT expression were downregulated. The changes mentioned above could be partly reversed by CTS. (*n* = 4–6 per group, one-way ANOVA, *p < 0.05 and **p < 0.01 compared to the control group; #*p* < 0.05 and ##*p* < 0.01 compared to the CP group)

RT-PCR assays were performed to determine the mRNA expression of TLR2, TLR4, CD14 NF-κB, and PPAR (Fig. 9a–e). Among them, the mRNA expression of TLR2, NF-κB, and PPAR was significantly higher in the CP group than in the control group, while the increase could be partly reversed after administration of CTS. In addition, levels of SOD, CAT, and GPx5 were detected (Fig. 9f–h). Reduction of antioxidant enzyme could be found in the CP group. Compared to the CP group, the CP plus CTS group showed an increase in level of SOD and CAT. Changes on gene expression of TLR/NF-κB, PPAR, and ROS were stimulated by ligature-induced periodontitis and could be prevented by CTS.

### Effects of ligature-induced periodontitis on the STAT3 signaling pathway

**Fig. 10:**
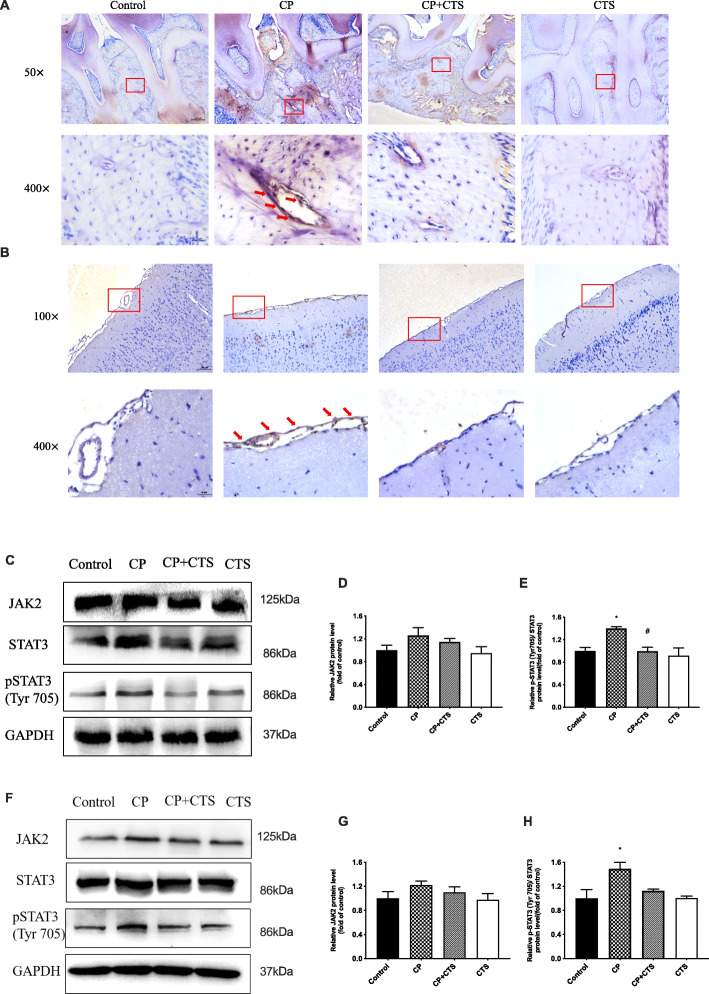
Effects of ligature-induced periodontitis on STAT3 signaling pathway. Expression of pSTAT3 in periodontal tissue (**a**) and cortex (**b**) was detected via immunohistochemistry (×100 and ×400, bar = 200 μm and 50 μm, respectively). Expression of JAK2, STAT3, and pSTAT3 (Tyr-705) was further measured by WB (**c**–**h**). High expression of pSTAT3 was effectively inhibited by CTS. pSTAT3/STAT3 was upregulated by the periodontal ligature. The quantification of related protein expression: **d** JAK2 in periodontal tissue (*F* = 1.800, *p* = 0.2251), **e** pSTAT3 (Tyr-705)/STAT3 in periodontal tissue (*F* = 6.685, *p* = 0.0143), **g** JAK2 in cortex (*F* = 1.361, *p* = 0.3224), **h** pSTAT3 (Tyr-705)/STAT3 in the cortex (*F* = 6.169, *p* = 0.0178). (*n* = 3 per group, one-way ANOVA, *p < 0.05 compared to the control group and #*p* < 0.05 compared to the CP group)

As shown in Fig. 10a,b, results of IHC showed an activation of pSTAT3 in oral cavity and cortex. Besides, WB was used to detect elevated expression of JAK2, STAT3, and pSTAT3 (Tyr705) in both periphery and the cortex of the CP group, which could be reduced by CTS (Fig. 10c–h). The results suggested that ligature-induced periodontitis could induce peripheral inflammation as well as neuroinflammation through activation of pSTAT3 pathway cascades.

### Effects of ligature-induced periodontitis on APP processing

**Fig. 11:**
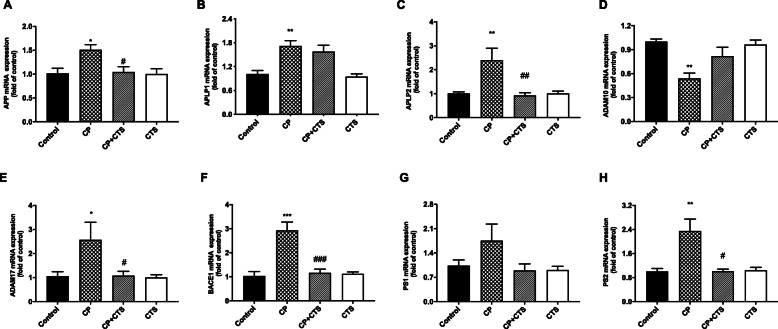
Effects of ligature-induced periodontitis on APP processing. RT-PCR was performed to detect mRNA expression of APP, APLP1, and APLP2 on genes in comparison to the control group, while these changes were reversed by CTS. The mRNA level of APP and the homologs in the cortex in each group: **a** APP (*n* = 4, *F* = 1.361, *p* = 0.3224), **b** APLP1 (*n* = 4, *F* = 1.361, *p* = 0.3224), **c** APLP2 (*n* = 6, *F* = 1.361, *p* = 0.3224). The mRNA level of secretases in the cortex in each group: **d** ADAM10 (*n* = 4, *F* = 1.361, *p* = 0.3224), **e** ADAM17 (*n* = 4, *F* = 1.361, *p* = 0.3224), **f** BACE1 (*n* = 6, *F* = 1.361, *p* = 0.3224), **g** PS1 (*n* = 4, *F* = 1.361, *p* = 0.3224), **h** PS2 (*n* = 6, *F* = 1.361, *p* = 0.3224). (*n* = 4–6 per group, one-way ANOVA, *p < 0.05, **p < 0.01, and ***p < 0.001 compared to the control group and #*p* < 0.05, ##*p* < 0.01, and ###*p* < 0.001 compared to the CP group)

As shown in Fig. 11, ligature-induced periodontitis modulated APP processing. RT-PCR was performed to detect increased expression of APP and two homologs, APLP1 and APLP2, especially APLP2, was found in the CP group (Fig. 11a–c). We also detected the mRNA levels of APP secretases, including α-, β-, and γ-secretases. After periodontal ligature, there were significant increases in the mRNA expression of ADAM17, BACE1, and PS2 and a decrease in ADAM10 mRNA expression in comparison to that in the control group (Fig. 11d–h). Changes mentioned above could be partly reversed by CTS.

### Effects of ligature-induced periodontitis on levels of Aβ

**Fig. 12:**
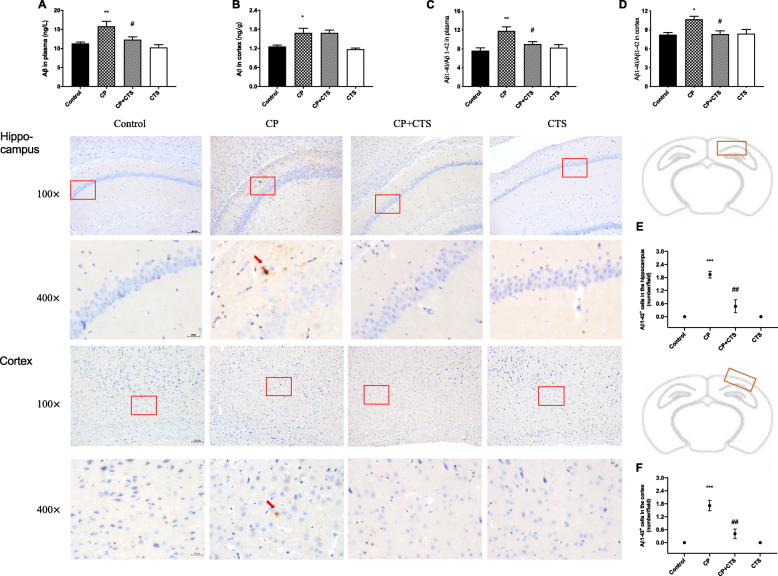
Effects of ligature-induced periodontitis on level of Aβ. ELISA was performed to detect the total level of Aβ and the Aβ ratio (Aβ1-40/Aβ1-42) in the plasma and cortex: **a** total level of Aβ in the plasma (*n* = 6, *F* = 8.239, *p* = 0.0009), **b** total level of Aβ in the cortex (*n* = 6, *F* = 8.778, *p* = 0.0006), **c** the Aβ ratio in the plasma (*n* = 4, *F* = 8.031, *p* = 0.0033), **d** the Aβ ratio in the cortex (*n* = 4, *F* = 5.304, *p* = 0.0147). Immunohistochemistry was used to conduct histopathological analysis of brain sections. Cytoplasmic yellow/brown cytoplasm was observed in both the hippocampus and cortex of the CP group. Quantification of Aβ1-42 levels was shown: **e** Aβ1-42 positive cells in the hippocampus (*n* = 3, *F* = 29.83, *p* = 0.0001), **f** Aβ1-42-positive cells in the cortex (*n* = 3, *F* = 24.53, *p* = 0.0002), (100× and 400×, bar = 200 μm and 50 μm, respectively). (*n* = 4–6 per group for ELISA and *n* = 3 per group for IHC analysis, one-way ANOVA, *p < 0.05, **p < 0.01, and ***p < 0.001 compared to the control group; #*p* < 0.05, ##*p* < 0.01, and ###*p* < 0.001 compared to the CP group)

According to ELISA, the total level of Aβ and the Aβ ratio (Aβ1-40/Aβ1-42) were increased in both the plasma and cortex in the CP group. The CP plus CTS group showed decreased production of Aβ in comparison to that in the CP group (Fig. 12a–d). In addition, IHC analysis was performed to assess expression of Aβ1-42. Representative images were shown in the hippocampal region and cortex, and the positive expression was increased in the CP group (Fig. 12e–f).

## Discussion

Ligature-induced periodontitis in rats triggered inflammatory status and could further lead to a decline in cognitive function. High expression of inflammatory cytokines, especially IL-6 and IL-21, was found both in the peripheral blood and cortex. Microglia and astrocytes were significantly activated in both the cortex and hippocampus. The STAT3 signaling pathway was activated in this progress, which further resulted in abnormal APP processing and cognitive impairment.

Although several studies found that periodontal pathogens can affect learning and memory ability, the effect of ligature-induced periodontitis on cognitive function has not yet been investigated [9, 10]. Periodontal ligature is a recognized and widely used method for establishing an animal model of periodontitis. As a promoting factor of plaque formation, ligature can cause continuous accumulation of plaques and infiltration of inflammatory cells, resulting in the destruction of periodontal connective tissue and loss of alveolar bone [22, 23]. Most studies used this model to evaluate the condition of the local periodontal tissue, while few focused on its impact on cognitive function [24, 25]. The results of our study showed that not only could periodontal ligature cause damage of periodontal tissues (Fig. 2), but also increase the levels of inflammatory cytokines in peripherial blood (Fig. 3) and cortex (Fig. 8). Besides, the learning and memory ability was impaired according to the MWM test (Fig. 5).

Dysregulated proinflammatory cytokines in periodontitis may induce inflammatory processes and neuroinflammation [7, 26]. Previous studies have shown that circulating IL-6 levels are obviously increased in patients with periodontitis [27]. In our study, we demonstrated that ligature-induced periodontitis can upregulate the levels of inflammatory factors, especially IL-6 and IL-21. The canonical IL-6 signaling pathway is initiated by the phosphorylation of STAT3 [28]. STAT3 is involved in the pathological process of the inflammatory response in the brain and plays a very important role [29–31]. Neuroinflammation is mainly caused by the activation of microglia and astrocytes and the release of cytokines, chemokines, or growth factors [32]. Both microglia and astrocytes are in a proinflammatory state in neurodegenerative disorders and neuroinflammation probably plays a substantial role in neurodegeneration [33]. This work demonstrated that ligature-induced periodontitis induced neuroinflammation via the activation of STAT3, as the levels of inflammatory factors were increased as well as the activated state of microglia and astrocytes were found in the hippocampus and cortex, while these changes could be alleviated by pSTAT3 inhibitor CTS (Figs. 6 and 7).

Previous studies have reported that effects of STAT3 inhibitor were mediated by the formation of ROS, and the role for STAT3 in the modulation of cellular ROS has been demonstrated [14, 15, 34]. Also, NF-κB regulates expression of a variety of genes involved in inflammation and it could induce generation of ROS [14]. Many researches indicated that changes of ROS (hypoxia/reoxygenation) promoted activation of NF-κB and STAT3 pathway [35–37]. Li et al. [38] reported that a ROS inhibitor could also downregulate levels of NF-κB and STAT3 pathways, the effect of which was similar to a NF-κB inhibitor. The STAT3 pathways, together with NF-κB pathways, are important signaling pathways involved in generation of ROS. Our results showed that CTS could decrease the levels of inflammation-related markers (such as TLR2, NF-κB, and PPAR) while significantly increase the levels of antioxidant enzymes including SOD and CAT (Fig. 9), which were similar to a previous study [39].

Besides, neuroinflammation could promote the progression of cognitive disorders such as Alzheimer’ disease (AD), for the increase of proinflammatory mediators and inflammatory cytokines secreted by activated microglia and astrocytes, all of which are known to contribute to Aβ production and accumulation [40, 41]. The STAT3 signaling pathway may be prevalent in such diseases, and multiple targets participate in the pathological process [42]. STAT3 can upregulate β-secretase (BACE1), which affects astrocyte metabolism and increases Aβ production [43]. After the Stat3 gene was knocked out of astrocytes, plaque deposition was reduced, while spatial learning and memory were restored [44]. Upregulation of APP and its homologs, as well as BACE1 and PS could be found in the present study, indicating that ligature-induced periodontitis could modulate APP processing through enhanced β- and γ-site secretase activity. Furthermore, levels of Aβ in the periphery and cortex were increased, and extracellular Aβ1-42 deposition could be found in the hippocampus and cortex. The periphery could generate and secret Aβ, which may explain the increase of Aβ1-40/Aβ1-42 in both the plasma and cortex. We proposed that continuous chronic peripheral and intrinsic inflammation, such as periodontitis and its chain reactions, could be a key feature of inflammatory pathology and attribute to abnormal APP processing and Aβ production (Figs. 11 and 12), similar to a previous study [45].

Consequently, we speculated that ligature-induced periodontitis can impair cognitive function, induce systemic inflammation and further neuroinflammation, which was represented by stimulation of microglia and astrocytes. Furthermore, it could modulate APP processing and increase the production of Aβ via the activation of STAT3 cascades. Our study showed that activation of the STAT3 by ligature-induced periodontitis could play an important role in the induction and amplification of neuroinflammation in cognitive impairment.

## Conclusions

In summary, we found that ligature-induced periodontitis could increase the inflammation load both in the periphery and the CNS, eventually led to cognitive impairment. In this process, STAT3 signaling pathway played an important role in promoting neuroinflammation and modulating APP processing. Thus, the activation of STAT3 cascades could be involved in the association between periodontitis and cognitive impairment.

## Supplementary Information

**Additional file 1:.** An example of negative controls in IHC was showed

## Data Availability

The datasets and materials supporting the conclusions of this article are included within the article.

## Abbreviations

Aβ: Amyloid β-protein
ADAM: A disintegrin and metalloproteinase
ANOVA: Analysis of variance
APP: β-amyloid precursor protein
APLP: Amyloid precursor-like protein
BCA: Bicinchoninic acid
BV/TV: Bone volume/total volume
BS/BV: Bone surface/bone volume
BMD: Bone mineral density
CAT: Catalase
CTS: Cryptotanshinone
DAB: Diaminobenzidine
ELISA: Enzyme-linked immunosorbent assay
GAPDH: Glyceraldehyde-3-phosphate dehydrogenase
GFAP: Glial fibrillary acidic protein
GPx5: Glutathione peroxidase 5
HE: Hematoxylin-eosin staining
Iba1: Ionized calcium-binding adaptor molecule 1
IHC: Immunohistochemistry
IL: Interleukin
JAK: Janus kinase
Micro-CT: Micro-computed tomography
NF-κB: Nuclear factor kappa-B
PBMCs: Peripheral blood mononuclear cells
PBS: Phosphate-buffered saline
PMSF: Phenylmethylsulfonyl fluoride
PPAR: Peroxisome proliferator-activated receptors
PS: Presenilin
PVDF: Polyvinylidene difluoride
RIPA: Radioimmunoprecipitation assay
SDS: Sodium dodecyl sulfate
SEM: Standard error of the mean
SOD: Superoxide dismutase
STAT: Signal transducer and activator of transcription
TLR: Toll-like receptor
WB: Western blot
