# Supplementary material for: Activated STAT3 signaling pathway by ligature-induced periodontitis could contribute to neuroinflammation and cognitive impairment in rats
Source: J Neuroinflammation. 2021 Mar 23;18:80. doi: 10.1186/s12974-021-02071-9 (PMC7986277; doi:10.1186/s12974-021-02071-9)
Supplement: Supplementary file 1 — Additional file 1:. An example of negative controls in IHC was showed [file 12974_2021_2071_MOESM1_ESM.docx]

Supplementary Material 1

Omission of the primary antibodies was used as negative controls in IHC.

For example, anti-phosphor STAT3-Tyr705 (9145S; Cell Signaling Technology, USA):


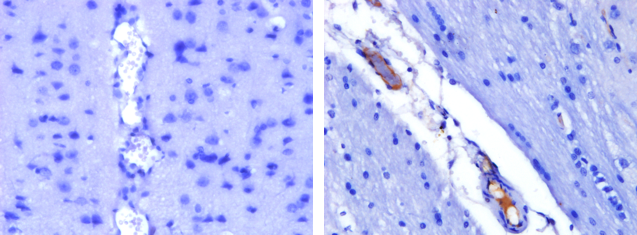
 (left: secondary-only control)
